# Bioactive Compounds and Antioxidant Activity of Red and White Wines Produced from Autochthonous Croatian Varieties: Effect of Moderate Consumption on Human Health

**DOI:** 10.3390/foods11121804

**Published:** 2022-06-19

**Authors:** Sanja Radeka, Sara Rossi, Ena Bestulić, Irena Budić-Leto, Karin Kovačević Ganić, Ivana Horvat, Igor Lukić, Fumica Orbanić, Teodora Zaninović Jurjević, Štefica Dvornik

**Affiliations:** 1 Institute of Agriculture and Tourism, Karla Huguesa 8, 52440 Poreč, Croatia; sarar@iptpo.hr (S.R.); ena@iptpo.hr (E.B.); ihorvat@iptpo.hr (I.H.); igor@iptpo.hr (I.L.); fumica@iptpo.hr (F.O.); 2 Institute for Adriatic Crops and Karst Reclamation, Put Duilova 11, 21000 Split, Croatia; irena.budic-leto@krs.hr; 3 Faculty of Food Technology and Biotechnology, University of Zagreb, Pierottijeva 6, 10000 Zagreb, Croatia; kkova@pbf.hr; 4 Clinical Hospital Centre Rijeka, Krešimirova ul. 42, 51000 Rijeka, Croatia; teodorazj@medri.uniri.hr (T.Z.J.); stefica.dvornik@medri.uniri.hr (Š.D.)

**Keywords:** autochthonous Croatian wines, bioactive compounds, moderate wine consumption, health

## Abstract

Moderate wine consumption is often associated with healthy lifestyle habits. The role of wine as a healthy drink is mainly due to its bioactive compounds, which differ according to various viticultural and enological factors. The aim of the present study was to observe the differences in bioactive compounds of white and red autochthonous Croatian wines, differing in terms of the grape variety and production technology. Our further aim was to explore the effect of their moderate consumption (200 mL per day) over the course of six weeks on some aspects of health in sixty-six healthy individuals. Participants were divided into eight groups depending on the wines consumed, while one group formed a non-consuming control group. Medical examination and laboratory tests were performed before the start and at the end of the consumption period. Systolic and diastolic blood pressure, total cholesterol, and LDL concentrations decreased. Additionally, an increase in HDL concentrations, and serotonin and dopamine levels, was observed. ALT, ALP, and GGT levels did not significantly increase in consumer groups, although alcohol concentration was relatively high in all the wines. Such results support the beneficial effects of wine-derived bioactive compounds on some health aspects resulting from moderate white and red wine consumption.

## 1. Introduction

Grapes and their products contain several major groups of bioactive compounds contributing to human health: phenolic acids, flavonoids, anthocyanins, proanthocyanidins, and stilbenes [1]. Moderate wine consumption of 200–300 mL/day is reported to have distinct health benefits, and is associated with a reduction in all-cause mortality [2,3]. Numerous studies provide evidence of the pharmacological, biological, and physiological benefits, including blood pressure, cholesterol, and lipid regulation; prevention of diabetes, obesity, atherosclerosis, cardiovascular, and neurodegenerative diseases; and anti-inflammatory and antitumor effects [4,5]. Additional to health benefits, consumers mostly consume wine because of its flavor, for food pairing, and for social interactions, at formal and special occasions [6]. In addition, Mortensen et al. [7] conclude that wine drinking is a general indicator of optimal social, cognitive, and personality development in Denmark.

Health effects of phenolic compounds are often attributed to their antioxidant activity, which is mediated by a variety of mechanisms, including the reduction or scavenging of reactive oxygen species, chelation of transition metal ions, and inhibition of enzymes involved in oxidative stress [8]. However, their influence on human health depends on the amount consumed and their bioavailability [9,10]. In the work conducted by Yoo Y.J. (2013) [11], the health-benefiting constituent is not sufficient to ensure certain health benefits from moderate drinking, while other authors [12,13] reported the positive effect of polyphenols in preventing certain diseases. The concentration of phenolic compounds in wine depends on various factors, such as variety, climate, soil, applied enological practices, ageing, and storage conditions [14]. Since the antioxidant activity of phenolic compounds depends on their chemical structure, the choice of enological practice is of great importance [15]. The maceration process, used mainly for red wines, has a positive effect on the antioxidant potential, and constitutes a fundamental difference in the vinification of red and white wine [16]. Red wines are considered to have a more protective effect on health than white wines, mainly because of the greater content of antioxidant substances released during the maceration process. As a result of grape processing in standard white and red wine production, phenolic compounds of white wines consist mostly of hydroxycinnamic acids and flavan-3-ols released from pulp cells, and the enzymatic oxidation products formed from them during pressing, while red wines contain large amounts of anthocyanins extracted from skins, and proanthocyanidins extracted from both skins and seeds [8]. Red wines usually contain up to a few grams of polyphenols per liter, while white wines have smaller concentrations of up to a few hundred milligrams, often below 0.3 mg/L [17]. In addition to phenols, macro- and microelements also contribute to the nutritional value of wine [18]; moreover, moderate daily wine consumption contributes to the essential element requirements of the human organism [16], while, on the contrary, some metals can be potentially toxic when consumed in excess [19]. Macro- and microelements play an essential role in the human body in terms of performing necessary functions, such as building strong bones and transmitting nerve impulses, and are able to participate in the biosynthesis of different hormones and regulate a normal heartbeat [20]. Although microelements have key roles in the formation of erythrocyte cells and the regulation of glucose levels, macroelements have a high potential to control blood pressure. Moreover, they are also involved in the immune and brain systems [20]. The maximum acceptable levels of metals in wine have been established by the International Organization of Vine and Wine [21], and, specifically for Croatia, similar rules are found in the national Official Gazette [22,23]. Their concentration in wines depends on many factors, such as the specific production location, as well as grape growing and winemaking conditions [24]. Among the nutrients required for the many physiologic functions essential to life are vitamins, which can also be found in wine. Vitamins are a group of highly complex compounds, organic in nature, present in foodstuffs in traces, and essential for normal metabolism [25]. They cannot be synthesized in the organism, and therefore their intake through diet is necessary [26]. Grapes contain many vitamins, and most of them are located in the grape skin, which is the reason red wines contain higher levels than white wines. The concentrations of vitamins initially present in grapes decrease during winemaking. Just few vitamins appear to be directly biologically active; as a result, metabolic conversion to another species or binding to a given protein is often necessary in order for a vitamin to become metabolically active [26]. Vitamin metabolic function concerns coenzyme activities in diverse pathways, reduction–oxidation systems, antioxidant activities, membrane integrity, cellular signaling, cellular protection, and yeast respiration [26,27].

The contact of wine with wooden barrels also modifies its composition [28]. Although primarily of grape origin, smaller amounts of phenolic compounds may be extracted from wood cooperage. Only trace amounts are derived from yeast metabolism [29]. Phenolic compounds undergo a number of reactions during maturation, aging, and storage [30], hence their content varies according to the maturation and storage conditions. The proportion of grape components in wine phenolic composition gradually decreases with ageing, as grape phenolics are converted to derived products. Continuous changes take place during winemaking and ageing, and additional compounds can be formed in barrel-aged wines [31]. The concentrations of flavonoids, flavan-3-ols, and cinnamic acids usually decrease with barrel ageing, but the increase in hydrolyzable tannins derived from oak wood leads to an increase in the total phenolic content of both white and red aged wines [32,33].

Several studies reported a high concentration of phenolic compounds in autochthonous Croatian wines, mainly because of the traditional processing of grapes in Croatia, which often includes prolonged maceration times, but also due to the inherent natural richness in polyphenols of certain Croatian varieties [34]. However, to our knowledge, there are a limited number of studies available that deal with the health properties of other non-nutritive or nutritive compounds present in wine, and their effect on human health resulting from the moderate consumption of autochthonous Croatian monovarietal wines produced by different vinification methods. In this study, autochthonous Croatian variety wines, young and aged, white (Malvazija istarska and Pošip) and red (Teran and Plavac mali) were investigated. The aim was to observe the differences in bioactive compounds of four white and four red autochthonous Croatian wines, which differ in terms of the grape variety and production technology, and to explore the effect of their moderate consumption, over the course of six weeks, on some aspects of health in healthy individuals.

## 2. Materials and Methods

### 2.1. Chemicals and Reagents for Wine Analysis

Folin–Ciocalteu reagent of analytical grade was supplied by BDH Prolabo (VWR, Leicester, UK). Hydrochloric acid was purchased from Carlo Erba (Rodano, Italy), sodium acetate and 2,2′-azobis(2-methylpropionamidine) dihydrochloride (AAPH) from Acros Organics, iron(III) chloride hexahydrate from POCh (Gliwice, Poland), TPTZ [2,4,6-tris(2-pyridyl)-s-triazine] from Alfa Aesar (Karlsruhe, Germany). Methanol, formic acid, water, and acetonitrile (all HPLC-grade purity), sodium dihydrogen phosphate, disodium hydrogen phosphate, fluorescein, 6-hydroxy-2,5,7,8-tetramethylchroman-2-carboxylic acid (Trolox), *trans*-caftaric acid, caffeic acid, syringic acid, quercetin hydrate, quercetin-3-glucoside *trans*-piceid, and vitamin standards were purchased from Sigma-Aldrich (Sigma-Aldrich, St. Louis, MO, USA). Gallic acid, protocatechuic acid, *p*-coumaric acid, ferulic acid, and taxifolin were from Fluka (Buchs, Switzerland); quercetin-3-glucuronide, procyanidins, (+)-catechin, (−)-epicatechin, piceatannol, and resveratrol were purchased from Extrasynthese (Genay, France); *p*-hydroxybenzoic acid, myricetin, and trifluoroacetic acid 99% (TFA) were from Acros Organics (Geel, Belgium). The *cis*-isomer of caftaric acid was obtained by UV illumination of a methanol solution containing the *trans*-isomer for four hours. Anthocyanins (monoglucoside chlorides) were from Biosynth Carbosynth (Bratislava, Slovakia), and *cis*-piceid solution was kindly donated by Urska Vrhovsek from Fondazione Edmund Mach. Ultrapure water, and all the reagents (60% HNO_3_) and standards for analysis using inductively coupled plasma optical emission spectroscopy (ICP-OES), were obtained from Merck (Darmstadt, Germany).

### 2.2. Chemicals and Reagents for Medical Analysis

For medical laboratory analysis, the biochemistry reagents used were supplied by Roche Diagnostic GmbH (Mannheim, Germany), intended for use on a Cobas 6000 analyzer (Roche Diagnostic GmbH, Mannheim, Germany); reagents supplied by Beckman Coulter (Germany) were for use on a biochemistry analyzer AU480 (Beckman Coulter, Krefeld, Germany). For the routine hematological tests, hematological reagents were obtained from Beckman Coulter (Brea, CA, USA) and were used on a hematological analyzer DxH 800 (Beckman Coulter, Brea, CA, USA). For the determination of serotonin and dopamine concentrations, reagents were purchased from Demeditec Diagnostics (Demeditec Diagnostics GmbH, Kiel, Germany).

### 2.3. Study Design, Subjects, and Materials and Methods

The study was conducted as part of the Croatian Scientific Foundation project “Influence of different vinification technologies on the qualitative characteristics of wines from Croatian autochthonous varieties: the role of wine in human diet”(acronym: VINUM SANUM), at the Institute of Agriculture and Tourism Poreč (Croatia). A total of 66 healthy participants, both men and women, between 22 and 65 years of age, were recruited for the study during June and July of 2020. Participants provided their informed consent and the study was carried out in accordance with the Declaration of Helsinki developed by the World Medical Association, and was approved by the Ethics Committee of the Clinical Hospital Centre Rijeka (Croatia).

Before conducting the study, participants were given a questionnaire to determine their drinking habits. Participants were divided into eight groups based on the type of wine consumed, while non-consumers (control group) formed a separate group. Based on the questionnaires, only those participants who never consumed wine and who consumed wine on special occasions or only several times a year were recruited for the control (non-consuming) group. The other participants that were divided into consumer groups, moderately consumed wine more frequently, from several times a month to every day, and, as such, were habitual drinkers. Each consumer group was given a single type of wine to consume. A total of eight types of wines were divided between the groups in a randomized system. Consumer groups of participants consumed 200 mL of wine daily during mealtimes for six weeks. All participants were asked to maintain their usual dietary habits during the consumption period, and to abstain from other alcoholic beverages except for wines provided by the project team, in the case of consumer group participants. The non-consuming group was not allowed to consume any kind of alcoholic beverage during the six-week period.

Medical examination and laboratory tests were conducted at the beginning and at the end of the six-week consumption period for all study participants. Medical examination included measuring participant’s weight, height, and waistline and hip width. Heart rate, and systolic and diastolic blood pressure, was measured, and an electrocardiogram (ECG) was obtained. Data regarding health, therapy, smoking, and alcohol consumption habits were recorded. Laboratory tests included routine biochemistry and hematology analysis and the determination of serotonin and dopamine concentrations.

For the purpose of conducting this study, a total of eight wines, four young and four aged, were collected from Croatian wine producers during 2019. Red autochthonous Croatian varieties Teran and Plavac mali, as well as white varieties Malvazija istarska and Pošip, were obtained from six local producers from different parts of coastal Croatian regions: Hrvatska Istra subregion (young and aged Teran, young and aged Malvazija istarska) and Central and Southern Dalmatia subregion (young and aged Plavac mali, young and aged Pošip). Only wines labeled with a protected designation of origin (PDO), and with a traditional term of Quality or Top-Quality, were selected. Both young white wines were produced in 2019 with a standard white wine production technique, without maceration, in stainless steel tanks. Aged white wines were produced in 2017, and their production included seven days of maceration, followed by ageing for 12–24 months in barrique barrels made from oak wood. Both young red wines were produced in 2019 by standard production practices for red wines, with 7–8 days of maceration in stainless steel tanks. Aged red wines, produced in 2017, went through a prolonged maceration process of 15–30 days, followed by ageing in barrique barrels for 1–2.5 years.

### 2.4. Standard Physico-Chemical Analysis

Each wine was analyzed in accordance with OIV methods [21], providing results for alcoholic strength by volume (%), reducing sugars (g/L), total dry extract (g/L), total dry extract without reducing sugars (g/L), total acidity (g/L), volatile acidity (g/L), and pH, as reported in Table 1 and Table 2.

### 2.5. Analysis of Total Phenolic Content and Antioxidant Capacity

Total phenolic content of each wine was determined by the Folin–Ciocalteu colorimetric method [35] using a Cary 50 UV/Vis spectrophotometer (Varian Inc., Harbour City, CA, USA). The absorbance was measured against a blank at a wavelength of 765 nm, and the results are expressed as gallic acid equivalents in mg/L of wine (mg GAE/L).

The antioxidant capacity of the wines was determined by the ferric reducing/antioxidant power (FRAP) assay, and the oxygen radical absorbance capacity (ORAC) assay. The FRAP assay was conducted according to the method of Benzie and Strain [36], and the results are expressed in mmol/L FeSO_4_ × 7H_2_O. The ORAC assay was performed according to Ninfali et al. [37], as briefly described in [38]. Fluorescence was measured by a Varian Cary Eclipse Spectrofluorometer (Palo Alto, CA, USA). The results were calculated as ORAC values using the difference in the area under the fluorescein decay curve between the blank and the sample. The results are expressed as mmol/L of Trolox equivalents (TE). Analyses of antioxidant capacity by FRAP and ORAC assays were conducted in triplicate.

### 2.6. Analysis of Individual Phenolic Compounds

Separation of individual phenolic compounds was carried out by high-performance liquid chromatography (HPLC) using an Agilent Infinity 1260 system (Agilent Technologies, Palo Alto, CA, USA) equipped with a quaternary pump, autosampler, column oven, and G4212B DAD and G7121B FLD detectors, applying several analysis methods. The wine samples were filtered through 0.45 μm-pore size PTFE filters prior to injection of 10 μL into the column. Column oven temperature was set at 26 °C, except for the analysis of anthocyanins (40 °C).

Hydroxycinnamic acids, hydroxybenzoic acids, taxifolin, flavonols, and flavonol glycosides were analyzed according to the modified method proposed by [39], using a reversed-phase Poroshell column 120 EC-C18 (150 × 4.6 mm i.d., particle size 2.7 μm, Agilent Technologies) equipped with a guard (Poroshell 120 EC-C18, 5 × 4.6 mm i.d., 2.7 μm, Agilent Technologies). Water/formic acid (99:1, *v*/*v*) (solvent A) and acetonitrile (solvent B) were used. The gradient conditions are described in our previous study [40]. Chromatograms were recorded at 280 nm (for hydroxybenzoic acids and taxifolin), 330 nm (for hydroxycinnamic acids), and 360 nm (for flavonols and flavonol glycosides).

Separation of flavan-3-ols was carried out according to the method proposed by Ćurko et al. [41] for grape skins, on a reversed-phase Zorbax SB-C18 column (250 × 4 mm i.d., particle size 5 μm, Agilent Technologies). Water/formic acid (99:1, *v*/*v*) (solvent A) and acetonitrile/formic acid (99:1, *v*/*v*) (solvent B) were used at a flow rate of 1 mL/min, under gradient conditions described by Rossi et al. [40]. Detection was performed using a fluorescence detector (FLD) set at 280 nm excitation and 320 nm emission with medium fluorescence intensity.

Analysis of stilbenes was carried out according to the method proposed by Mark et al. [42], using the same Zorbax SB-C18 column. Methanol/water/acetic acid (10:90:1, *v*/*v*) (solvent A) and methanol/water/acetic acid (90:10:1, *v*/*v*) (solvent B) were used at a flow rate of 1.5 mL/min. Chromatograms were recorded at 306 nm.

Anthocyanins were separated according to the modified OIV method [43] using a Poroshell 120 EC-C18 column and a guard, as mentioned earlier (Agilent Technologies, Palo Alto, CA, USA). Water/formic acid/acetonitrile (87:10:3, *v*/*v*/*v*) (solvent A) and water/formic acid/acetonitrile (40:10:50, *v*/*v*/*v*) (solvent B) were used at a flow rate of 0.7 mL/min. The gradient conditions were: 0 min, 6% B; 13.6 min, 30% B; 27.21 min, 50% B; 31.74 min, 60% B; 37.18 min, 6% B; 40.81 min, 6% B. Chromatograms were recorded at 518 nm.

Identification was performed by comparing retention times and/or UV/Vis spectra with those of pure standards when available. Quantification was carried out using standard calibration curves. For *cis*-caftaric acid and *cis*-piceid, for which only qualitative standards were available, semi-quantitative analysis was carried out. Acetyl and *p*-coumaroyl derivates of anthocyanins were identified by comparing retention times reported in the characteristic chromatogram in the OIV method [43] and quantified using a standard curve of corresponding anthocyanins.

### 2.7. Inductively Coupled Plasma Optical Emission Spectroscopy (ICP-OES) Analysis for Macro- and Microelements in Wine

The determination of macro- and microelements was conducted using an Optima DV 2000 inductively coupled plasma optical emission spectrometer (Perkin Elmer, Shelton, CT, USA) equipped with a Meinhard spray chamber, nebulizer, and peristaltic sample delivery system. The samples were introduced into the plasma under operating conditions [44] and following the procedure previously described by Rossi et al. [40]. Both calibration solutions and wine samples with ethanol removed [45] were analyzed, based on the proposed method, in 2% HNO_3_. Analyzed elements were identified in line with ICP-OES protocol using the WinLab 1.35 Perkin Elmer software, and quantified by direct calibration method.

### 2.8. Analysis of Vitamins in Wine

Chromatographic analyses were performed on an Agilent 1100 Series liquid chromatography system (Agilent Technologies, Waldbronn, Germany) with a DAD and a single quadrupole mass detector equipped with an electrospray ionization interface (G1946D). Separation of vitamins was performed on a Luna Phenomenex C18 (5 μm, 150 × 4.6 mm) column at room temperature following the protocol of Trang et al. [46]. The mobile phase consisted of two solvents: 0.025% TFA in water (solvent A) and acetonitrile (solvent B). Flow rate was set at 1 mL/min. Detection was performed in the range of 190–400 nm. MS analysis was performed using 0.1% formic acid (solvent A) and acetonitrile (solvent B). The injected volume was 10 µL and the flow rate was set at 0.6 mL/min. The mass parameters were as follows: capillary voltage 4000 V, drying gas temperature 350 °C, gas flow (N_2_) 12 mL/min, operated in positive ion mode. Identification of five vitamins (B1, B2, B3, B6, and C) was carried out by comparison with the retention times of authentic standards and their spectral properties, respectively. Identified compounds were quantified by direct calibration method.

### 2.9. Medical Examination and Laboratory Tests

Blood samples from all participants were collected into vacuum tubes (Becton Dickenson Company, Franklin Lakes, NJ, USA) and analyzed according to standard and dopamine analysis.

A 12-lead resting electrocardiogram (ECG) was recorded using a Cardiofax S (Nihon Kohden, Tokyo, Japan) machine. The ECG analysis was interpreted by the investigator. Body mass index (BMI) was calculated as kg/m^2^.

### 2.10. Statistical Data Analysis

To observe the differences between the various chemical parameters of the wines, the data were subjected to one-way analysis of variance (ANOVA), and average values were compared using Fisher’s Least Significant Difference (LSD) test at the level of *p* < 0.05. Pearson’s correlation coefficient was calculated. The software used was Statistica v.13.2 (Stat-Soft Inc., Tulsa, OK, USA).

To investigate the differences in the changes in the medical examination and laboratory test parameters between the first and the second measurement among the different groups of consumers and the non-consumer group, the data for one-way analysis of variance (ANOVA) were obtained by subtracting the values of the two measurements for each parameter. In this way, a comparison of the relative changes was enabled, and differences due to different initial absolute values were eliminated.

## 3. Results and Discussion

### 3.1. Standard Physico-Chemical Analysis

The results of the standard physico-chemical analysis of both red and white wines are reported in Table 1 and Table 2. The volume fraction of alcohol in analyzed wines ranged from 12.99 to 14.16 %vol. When observing white wines, significantly the highest alcohol content was noted in aged Pošip wines that contained 14.16 ± 0.02 %vol., while the lowest alcoholic strength was found in young Malvazija istarska wines, containing 12.99 ± 0.01 %vol.

**Table 1:** Concentrations of standard physico-chemical analysis parameters of young and aged Malvazija istarska and Pošip wines.

Physico-Chemical Parameters	Malvazija Istarska		Pošip	
	Young	Aged	Young	Aged
Alcoholic strength (%vol.)	12.99 ± 0.010 ^d^	13.99 ± 0.010 ^b^	13.11 ± 0.020 ^c^	14.16 ± 0.020 ^a^
Total dry extract (g/L)	21.8 ± 0.0 ^ab^	21.5 ± 0.64 ^b^	20.6 ± 0.10 ^c^	22.1 ± 0.10 ^a^
Reducing sugars (g/L)	2.3 ± 0.1 ^a^	2.1 ± 0.0 ^b^	1.8 ± 0.1 ^c^	2.3 ± 0.0 ^a^
Extract without sugars (g/L)	18.5 ± 0.06 ^a^	18.4 ± 0.64 ^a^	17.7 ± 0.06 ^b^	18.8 ± 0.10 ^a^
Ash (g/L)	2.83 ± 0.01 ^a^	2.66 ± 0.02 ^b^	1.80 ± 0.01 ^d^	1.86 ± 0.01 ^c^
pH	3.63 ± 0.01 ^a^	3.34 ± 0.01 ^b^	3.20 ± 0.01 ^c^	3.07 ± 0.00 ^d^
Total acidity (g/L) ^1^	5.0 ± 0.01 ^d^	5.6 ± 0.01 ^c^	5.8 ± 0.02 ^b^	7.0 ± 0.01 ^a^
Volatile acidity (g/L) ^2^	0.59 ± 0.01 ^a^	0.44 ± 0.00 ^c^	0.39 ± 0.01 ^d^	0.56 ± 0.01 ^b^

^1^ As tartaric acid. ^2^ As acetic acid. Each value represents the mean ± standard deviation; young white wines produced without maceration and aged white wines produced with 7 days maceration followed by maturation in oak barrels. Different lowercase superscript letters represent statistically significant differences between wine samples at *p* < 0.05 obtained by one-way ANOVA and least significant difference (LSD) test.

The alcohol content in red wines also varied among samples, with aged Teran wine having significantly the highest (13.79 ± 0.01 %vol.) and young Plavac mali the lowest (13.18 ± 0.02 %vol.) alcoholic strength.

**Table 2:** Concentrations of standard physico-chemical analysis parameters of young and aged Teran and Plavac mali wines.

Physico-Chemical Parameters	Teran		Plavac Mali	
	Young	Aged	Young	Aged
Alcoholic strength (%vol.)	13.20 ± 0.010 ^c^	13.79 ± 0.010 ^a^	13.18 ± 0.020 ^d^	13.57 ± 0.010 ^b^
Total dry extract (g/L)	29.1 ± 0.1 ^d^	34.5 ± 0.1 ^a^	29.8 ± 0.1 ^c^	33.6 ± 0.0 ^b^
Reducing sugars (g/L)	2.6 ± 0.3 ^b^	2.2 ± 0.2 ^c^	1.8 ± 0.1 ^d^	3.1 ± 0.2 ^a^
Extract without sugars (g/L)	25.5 ± 0.06 ^d^	31.3 ± 0.06 ^a^	26.9 ± 0.06 ^c^	29.5 ± 0.00 ^b^
Ash (g/L)	2.70 ± 0.01 ^d^	4.04 ± 0.02 ^a^	3.86 ± 0.01 ^b^	3.34 ± 0.01 ^c^
pH	3.42 ± 0.01 ^d^	3.61 ± 0.01 ^c^	3.95 ± 0.00 ^a^	3.71 ± 0.00 ^b^
Total acidity (g/L) ^1^	6.0 ± 0.1 ^b^	6.5 ± 0.0 ^a^	4.3 ± 0.1 ^d^	5.3 ± 0.0 ^c^
Volatile acidity (g/L) ^2^	0.44 ± 0.01 ^c^	0.88 ± 0.01 ^a^	0.37 ± 0.01 ^d^	0.6 ± 0.0 ^b^

^1^ As tartaric acid. ^2^ As acetic acid. Each value represents the mean ± standard deviation; young red wines produced with standard 7–8 days maceration and aged red wines produced with prolonged maceration (15–30 days) and maturation in oak barrels. Different lowercase superscript letters represent statistically significant differences between wine samples at *p* < 0.05 obtained by one-way ANOVA and least significant difference (LSD) test.

All the wines were dry with < 4 g/L of residual sugars. Young Pošip had significantly the lowest extract without sugar content in relation to the other white wines, which did not differ significantly between each other, while ash content and pH were significantly highest in young Malvazija istarska white wine. Among red wines, aged Teran wine had significantly the highest content of extract without sugars and ash, and young Plavac mali had the highest pH value. Young Teran was characterized by a significantly lower concentration of extract without sugar and ash content in relation to the other red wines. The total acidity values varied between 5.0 to 7.0 g/L in white wines, being significantly highest in aged Pošip wines and lowest in young Malvazija istarska wines, and from 5.3 to 6.5 g/L in red wines, with the highest values observed in aged Teran wines and the lowest in young Plavac mali wines. Such acidity values for Pošip wines were reported in an earlier study [47], while high total acidity levels in Teran wines were previously reported by [40]. Volatile acidity concentration varied from 0.39 ± 0.01 to 0.59 ± 0.01 mg/L in white wines and 0.37 ± 0.01 to 0.88 ± 0.01 mg/L in red wines, which is in accordance with the values provided by the Croatian Regulation on Wine Production [22].

### 3.2. Total Phenolic Content and Antioxidant Capacity

**Figure 1:**
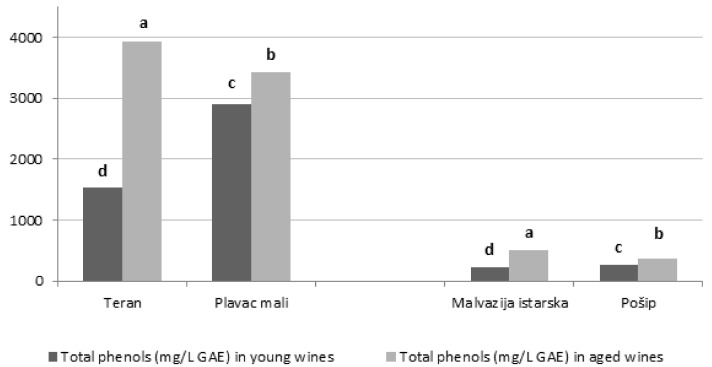
Total phenolic content (TPC) of young and aged Croatian wines; red—Teran and Plavac mali, and white—Malvazija istarska and Pošip: young white wines produced without maceration, young red wines produced with standard 7–8 days maceration, aged wines produced with maceration of 7 days (white wines) and prolonged 15–30 days (red wines) followed by maturation in oak barrels. Each value represents the mean ± standard deviation; different lowercase letters represent statistically significant differences between wine samples at *p* < 0.05 obtained by one-way ANOVA and least significant difference (LSD) test.

**Figure 2:**
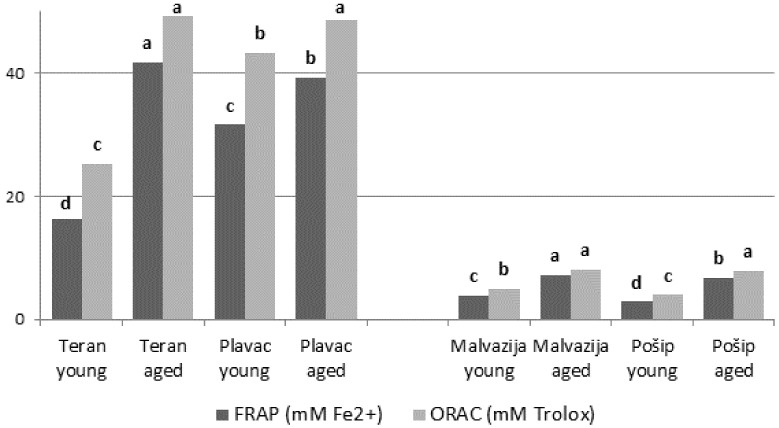
Antioxidant activity (FRAP and ORAC) of young and aged Croatian wines; red—Teran and Plavac mali, and white—Malvazija istarska and Pošip: young white wines produced without maceration, young red wines produced with standard 7–8 days maceration, aged wines produced with maceration of 7 days (white wines) and prolonged 15–30 days (red wines) followed by maturation in oak barrels. Each value represents the mean ± standard deviation; different lowercase letters represent statistically significant differences between wine samples at *p* < 0.05 obtained by one-way ANOVA and least significant difference (LSD) test.

The results of total phenolic content and antioxidant capacity measured using two different antioxidant assays (FRAP and ORAC) are presented in Figure 1 and Figure 2, respectively. Total phenolic content varied from 1527.12 to 3936.21 mg/L in red wines, and 226.2 to 505.4 mg/L in white wines; such differences between red and white varieties are in accordance with previously published papers [48,49]. Similar results were reported by Yoo et al. [50]; their study showed that baseline levels of total phenols in red wines are more than five times higher than those of white wines. In addition, higher total phenolic content in aged white and red wines was probably mainly due to the increase in the content of hydrolyzable tannins derived from oak, as reported earlier [32,33]. However, it is important to emphasize that white wines aged in barrels underwent the maceration process for 7 days, while the maceration for red barrel-aged wines lasted from 15 to 30 days; hence, their phenolic content was probably already higher than that of young wines even prior to aging. According to [34,51], the reason for higher total phenolic content in red in relation to white wines is a result of the higher content of condensed tannins and the presence of anthocyanins, and as a consequence of better extraction of phenolic compounds from grape pomace during fermentation of the juice on the skins and seeds. In a study conducted by Rastija et al. [49] the differences in total phenolic content between wines of different wine-growing subregions of Croatia were also observed. These authors noted that young wines from the Central and Southern Dalmatia subregion were more abundant in total phenols than wines from the Hrvatska Istra subregion. Such results are in agreement with those of our study: the wines from the Central and Southern Dalmatia subregion (red Plavac mali and white Pošip wine) exhibited significantly the highest total phenolic content, however, only in young wines; conversely, when observing aged wines in our study, wines from the Hrvatska Istra subregion (red Teran and white Malvazija istarska wine) exhibited significantly the highest values.

The highest concentration of total phenolic content among all the red wines was observed in aged Teran wines, being 2.5-fold higher than in young Teran wine. Aged Malvazija istarska exhibited significantly the highest total phenolic concentrations among all the white wines, two-fold higher than those observed in young Malvazija istarska wines.

The antioxidant capacity of white and red monovarietal wines varied significantly, which is in accordance with Vrček et al. [16]. When comparing the two assays, ORAC resulted in higher values than the FRAP assay; however, a strong correlation was noted between the two assays (r = 0.992). According to the results obtained by FRAP assay for red wines, aged Teran wines had significantly the highest antioxidant capacity, while in the ORAC assay, both aged Teran and aged Plavac mali exhibited the highest values in relation to the other wines. Significantly, the lowest antioxidant capacity was observed in young Teran wines in both assays. The increase in antioxidant capacity also varied according to the wine maturation process. Antioxidant capacity of aged Teran wine was 155% (FRAP) and 95% (ORAC) higher than that of young Teran, which was not aged in an oak barrel. In the case of Plavac mali wines, these differences were lower (24% in FRAP and 13% in ORAC assay, respectively). Among white wines, the FRAP assay indicated significantly the highest values in aged Malvazija istarska wines, and the lowest values in young Pošip wines. Both aged Malvazija istarska and aged Pošip wines exhibited the highest values according to the ORAC assay, and the lowest antioxidant capacity values were observed in young Pošip wines. When observing the antioxidant capacity between young and aged wines, aged Malvazija istarska showed 84% (FRAP) and 64% (ORAC) higher antioxidant capacity than young Malvazija istarska. For the Pošip variety, the aged Pošip wine showed 138% and 99% higher antioxidant capacity than the young Pošip wine according to FRAP and ORAC assays, respectively.

Total phenolic content of red wines correlated strongly with both FRAP and ORAC values, with the coefficients r = 0.982 and r = 0.934, respectively. A slightly weaker correlation strength was observed for total phenolic content of white wines and FRAP and ORAC antioxidant capacity values, with the coefficients r = 0.863 and r = 0.849, respectively. A high correlation of total phenolic content and antioxidant activity was noted in earlier studies [52], confirming that the source of antioxidant activity derives from phenolic compounds in wine [48].

### 3.3. Individual Phenolic Compounds

Four white and four red wines were compared separately on the basis of their phenolic content and composition. For concentrations of nearly all of the investigated phenols, significant differences were found (Table 3 and Table 4). The sum of total HPLC phenols in white wines was significantly higher for aged wines that went through the maturation process in oak barrels, than for young white wines (Table 3). This was also determined for red wines, and could be due to extraction from wooden barrels during wine aging [29], as well as resulting from maceration, as stated previously. Total HPLC phenols in white wines were significantly highest in aged Pošip, and significantly lowest in young Pošip wine. When observing the differences between the varieties, aged Pošip contained significantly higher total HPLC phenol values than aged Malvazija, and, in contrast, young Malvazija contained significantly more HPLC phenols in total than young Pošip. The same relationship among white wines was established according to total phenolic acid content, which made up the majority of the total phenols.

Total HPLC phenols in white wines were significantly the highest in aged Pošip, and significantly the lowest in young Pošip wine. When observing the differences between the varieties, aged Pošip contained significantly higher total HPLC phenol values than aged Malvazija, and, in contrast, young Malvazija contained significantly more HPLC phenols in total than young Pošip. The same relationship among white wines was established according to total phenolic acid content, which made up the majority of the total phenols. Regarding red wines, total HPLC phenols were significantly the highest in aged Teran, and significantly the lowest in young Teran wine (Table 4). Aged Teran contained significantly more HPLC phenols in total than aged Plavac mali and young Plavac mali, and significantly higher values than young Teran. Red wines were also characterized by a high content of phenolic acids, and only in young Plavac was the total flavan-3-ol concentration higher than the total phenolic acid concentration. Young Pošip contained higher concentrations of hydroxycinnamic acids compared to young Malvazija, which was previously shown to be characteristic in the comparison of these varieties [53]. This difference was, in the present study, also determined for aged wines. Gallic acid was prevalent among the determined hydroxybenzoic acids, which was previously reported for both white and red wines [53,54,55].

Among hydroxycinnamates, *trans*-caftaric acid was the most abundant in red wines, which is in accordance with the findings of Lukić et al. [53], while in white wines, caffeic acid was the most predominant, reaching highest concentrations in aged Pošip wine. Caffeic acid concentration in young Malvazija istarska wine was 9.6-fold higher than in young Pošip wine; such caffeic acid concentrations in young, non-macerated Malvazija wines are similar to those previously reported by Bestulić et al. [56].

Total flavan-3-ol concentration was significantly highest in young Pošip. In young wine, it was significantly higher than in aged wine of the same variety. Among red wines, aged wines had significantly higher amounts of flavan-3-ols than young wines. Aged Teran had the highest total phenolic acid, total flavan-3-ol, and total stilbene concentrations, followed by aged Plavac mali wine. It is worth mentioning that red wines contained noticeably higher concentrations of quercetin, and total flavonol concentration was highest in aged Teran, followed by young Plavac mali wine. Pošip wines had higher total stilbene content, the highest being found in aged Pošip. Young and aged Malvazija istarska wines did not differ significantly when compared at the total stilbenes level. A previous study by Lukić et al. [53] reported the same characteristic difference in concentrations of total stilbenes between Pošip and Malvazija istarska wines.

Total anthocyanin content was highest in Plavac mali wines. Lower concentrations of anthocyanins were found in aged red wines. Similar results were reported by Pérez-Trujillo et al. [57] for Spanish red wine varieties. According to the literature, the concentration of monomeric anthocyanins in wine constantly declines. These compounds are involved in a wide variety of reactions during fermentation and aging, resulting in the formation of new red pigments (anthocyanin–tannin adducts and pyranoanthocyanins), or leading to their disappearance due to the oxidation and precipitation of polymeric matter [29,51].

### 3.4. Macro- and Microelements

In the wines investigated in this study, the following macro- and microelements were identified: K, Ca, Mg, and Na as macroelements, and Al, Cu, Fe, and Mn as microelements. Their concentrations are reported in Table 5 and Table 6. Significantly, the highest concentrations of total macroelements, regarding white wines, were detected in young Pošip wines (Table 5), while regarding red wines, these concentrations were significantly highest in young and aged Teran wines (Table 6). Significantly, the highest concentrations of total microelements, regarding white wines, were detected in young Pošip and young Malvazija istarska wines, while regarding red wines, these concentrations were significantly highest in young Teran wines. It is important to note that the concentration of total microelements in red wines was almost four times higher when compared with white wines, which is in correspondence with the results of Rossi et al. [40], who concluded that longer maceration time significantly increased the concentration of total microelements in red Teran wines.

In the present study, the highest level of potassium (K) was detected in young Pošip among white wines, while in red wines, Plavac mali young and aged wines exhibited significantly highest concentrations. Several factors affect the amount of K in wine, including grape variety, soil and climatic conditions, time of harvest, temperature of fermentation, storage, and pH value. As concluded by Karatas et al. [58], a high level of K in wine has great nutritional value. The highest concentration of sodium (Na) was determined in young Malvazija istarska among white wines, and in aged Teran among red wines. A high proportion of K to Na in wine is considered a positive feature that could recommend moderate wine consumption [29]. The concentrations of calcium (Ca) were significantly highest in young Pošip wines among white, and in young Teran among red wines. Regarding magnesium (Mg) in the investigated white wines, the significantly highest concentration was detected in aged Pošip wines, while among red wines, the aged Teran wine contained the highest concentration. The Mg content in wines can be attributed to a number of factors, including the soil composition, pH, storage duration and temperature, and the rate of pressing. As reported by [40], significant increases in Mg concentration are affected by maceration duration, and this could explain the higher levels of Mg in all the investigated red wines when compared to white wines.

In all the investigated wines in this study, the concentrations of aluminum (Al) and copper (Cu) were detected in traces, and well under the concentration of 10 mg/L, which has been established by the International Organization of Vine and Wine [21] and Official Gazette [22,23] as the maximum limit allowed in wine. Manganese (Mn) levels in the investigated wines were generally low. As reported by [59], seeds contain three times as much manganese as skins, and thirty times as much as grape flesh, which can explain higher manganese concentrations in red wines. The concentration of Fe was the highest among all the investigated microelements, and the values were higher in red wines, with the significantly highest concentration in young Teran wines. However, Fe concentrations in the present study were below the maximum permitted limits in wine (10 mg/L) according to the Official Gazette [22,23]. The higher levels of Fe in red wines could turn out to be very important from a nutritional standpoint because it is well known that Fe is an essential element for almost all living organisms, where it participates in a wide variety of metabolic processes [60].

### 3.5. Vitamins

The following water-soluble vitamins were identified in wines investigated in this study: vitamin B1 (thiamine), vitamin B2 (riboflavin), vitamin B3 (niacin), and vitamin B6 (pyridoxine) (Table 7 and Table 8). The content of total B-complex vitamins was significantly highest in young Teran wines among red wines, and in young Malvazija istarska wines among white wines. When comparing the white and red wines investigated in this study, the total B-complex vitamin concentration was much higher in red wines, probably because the vitamins are mostly located in the grape skin.

In an earlier study, Velić et al. [61] reported that vitamin B1 is utilized by yeast during fermentation, so its levels in wine are insignificant. Moreover, they concluded that thiamine levels are lowered by reaction with SO_2_ during fermentation and absorption by bentonite. These results correspond with vitamin B1 levels in wines investigated in this study, where concentrations were generally low, and much lower in white wines in comparison with red wines. In addition, Evers et al. [62] reported that red wines appear to have higher thiamine concentrations than white wines, indicating that skins and seeds are richer in this compound than pulp and juice are. Concentrations of vitamin B2 were significantly highest in young Malvazija istarska among white wines, and in young Teran among red wines. Evers et al. [62] reported that it is highly probable that riboflavin in musts and wines results from yeasts rather than from the solid parts of grapes. It is important to note that in all the investigated wines, the concentration of vitamin B2 was much lower in aged wines compared with young wines; this is in agreement with results of Moreno and Peinado [63], who concluded that the photosensitivity of riboflavin may often lead to rapid depletions in its levels in wine with aging.

The concentrations of vitamin B3 were significantly highest in young Malvazija istarska among white wines, and young Teran among red wines. Evers et al. [62] reported that wines from red grape varieties display higher contents of niacin than white ones, which is also the case in this study. The concentrations of vitamin B6 were significantly highest in young Malvazija istarska among white wines, and young Teran among red wines. Hall et al. [64] concluded that the amounts of vitamin B6 in grapes differ, and are lower in white grape varieties when compared to red grape varieties, and that the same trend was noticed in wines. The same was observed in the present study. Furthermore, the same group of the above-mentioned authors found that levels of vitamin B6 show significant losses during fermentation. Vitamin C was not detected in any of the investigated wines. The stability of vitamins is often at risk due to the various technological practices used during processing, especially changes in temperature regimes and oxygen levels [65]; moreover, the concentration of vitamins decreases during fermentation and ageing. Velić et al. [61] reported that different environmental conditions, under which a plant is grown, such as temperature, water availability, pathogenic attack, and nutrients, affect the concentration of vitamin C.

### 3.6. Medical Examination and Laboratory Results

The results expressed as the differences in medical examination and laboratory test parameter values after the consumption period (six weeks) in eight consumer groups and one non-consumer group of the study participants are shown in Table 9 and Table 10. The measurements of weight, BMI, waistline, hip width, heart rate, and blood pressure (systolic and diastolic) were recorded before and after the consumption process. There were no significant differences in the changes in hip width, hearth rate, or diastolic blood pressure between groups. A decrease in body weight and BMI was recorded in the control group, and in young Pošip- and aged Plavac mali-consumer groups. An increase in weight and higher BMI were recorded after young Malvazija istarska and, especially, after aged Pošip consumption. Although all the wines had similar sugar concentration, aged Pošip wine had the highest alcohol concentration, so it could be assumed that the volume fraction of alcohol (rather than sugar concentration) in wine might have had a negative impact on body weight and BMI. There were no significant differences in hip width in participants between any of the study groups, although a small increase in hip width was noted at the end of the examination in all groups, as well as in the control group.

Hearth rate increased after young Pošip and young and aged Plavac mali wine consumption, while other consumption groups showed a decrease, although the changes between groups were not significant. According to Chiva-Blanch et al. [66], dealcoholized red wine decreases systolic and diastolic blood pressure, whereas moderate alcohol consumption in general does not affect blood pressure. As concluded by Guilford and Pezzuto [67], polyphenols are responsible for the health benefits of wine, rather than alcohol. In our study, systolic blood pressure mostly decreased after aged Plavac mali wine consumption, but it only significantly differed from the changes noted after young Malvazija istarska and aged Teran wine consumption.

When observing the laboratory test parameters, there were no significant differences in the changes in erythrocytes, glucose, eGFR, K, bilirubin, AST, iron, UIBC, feritin, HDL, and triglyceride concentrations between groups. The concentration of hemoglobin differed only between the aged Pošip group (in which the concentration of hemoglobin increased) and young Teran wine, which showed a decreasing trend. The most common cause of low hemoglobin levels in the blood is iron deficiency; however, we did not record a statistically significant decrease in iron concentration in any of the wine consumer groups. However, aged Pošip, young and aged Teran, and young Plavac mali groups showed a decrease in iron concentration. According to Ioannou et al. [68], moderate consumption of alcohol is associated with a reduction in the risk of iron deficiency. However, in cases of iron overload, it is recommended that one consumes wine with higher phenolic content because phenolics inhibit food iron absorption [69]. Although there were some differences in urea and creatinine concentrations in some examination groups at the end of study, statistically significant differences in eGFR were not recorded, showing that there were no significant changes in kidney function.

In young Malvazija and Plavac mali consumers, a significant increase in urea concentration was found at the end of study in relation to the control group, and young Pošip and young Teran consumer groups. Assuming it is impossible to connect higher urea concentrations with protein metabolism or kidney function, one of the possible explanations can be dehydration connected to wine consumption due to the presence of ethanol and its diuretic effects [70]. Although there was no linear correlation, the highest increases in urea and sodium concentrations were found in Plavac mali young wine consumers.

After consumption of most wines, the liver enzymes ALT, ALP, and GGT did not significantly increase in relation to the control group, although alcohol level was relatively high in all the wines (12.99 vol%–14.16 vol%). The rise in catalytic concentrations of ALT noted after young Plavac mali consumption differed from the other treatments, with the exception of aged Pošip and aged Teran groups. A similar trend was observed in the catalytic concentrations of GGT, which was highest in young Plavac mali and aged Teran groups.

There were no significant differences in triglycerides and HDL concentrations, before and at the end of the study, in any of the examination groups. Cholesterol concentrations decreased in almost all the examination groups except in aged Pošip and young and aged Teran groups, where a modest rise was recorded. In all these groups, almost the same increase in HDL concentration was recorded, so it was assumed that the observed increase in total cholesterol was related to HDL concentration (“good” cholesterol), rather than to LDL (“bad” cholesterol). LDL concentrations were higher only in aged Pošip and young Teran consumer groups, while the lowest LDL concentrations were observed after aged Plavac mali wine consumption. Additionally, it is important to note that aged Plavac mali wine consumers had mostly decreased systolic and diastolic blood pressure and total cholesterol concentration. Although the changes in HDL concentrations among groups were not significant, it is important to emphasize that an increasing trend was observed in each group. According to [71,72], moderate alcohol consumption is associated with higher levels of HDL, which was also evident in this study.

Serotonin and dopamine (“happiness” hormones) are the most important neurotransmitters, and they are involved in mood changes. Positive and negative moods are mediated by dopamine and serotonin levels [73]. Negative mood is associated with lower dopamine levels, and increased serotonin levels are related to positive mood [74,75]. In this study, consumption of some wines had a positive effect on the increase in serotonin and dopamine concentration in blood; on the other hand, consumption of some wines led to a decrease in these concentrations. White wines had a mostly positive impact on serotonin concentration, with the exception of the aged Pošip wine group, where a decrease was recorded. Regarding red wines, only young Plavac mali had a positive impact on blood serotonin concentrations. Consumers of aged Malvazija istarska wine exhibited the highest increase in serotonin blood concentration (16-fold higher compared to control group), while aged Plavac mali wine consumers had the highest decrease in serotonin blood concentration. The highest rise in blood dopamine concentration was recorded after young Malvazija istarska wine consumption among white wines (11-fold higher compared to control group) and aged Teran wine consumption among red wines (2.5-fold higher compared to control group).

Although both serotonin and dopamine are responsible for positive mood, they function in different ways, and, coded by specific genes and genetic factors, they have clear and significant effects on happiness [76]. Based on the results of the present study and previous studies, it is possible that different wine compositions can lead to such effects. It is known that white wines are generally of lighter structures, possess prominent fruity-floral aromas, and are less acidic, which can ultimately result in a greater appeal of these wines to consumers.

However, it is important to emphasize that the effects of moderate consumption on certain health aspects in this study were short-term, as the data were collected only over the study period of six weeks.

## 4. Conclusions

In this study, we observed that participants who consumed moderate amounts of wine produced from Croatian autochthonous grape varieties over six weeks experienced favorable effects concerning the decrease in systolic and diastolic blood pressure, and total cholesterol and LDL (‘’bad’’ cholesterol) concentration. Conversely, an increase in HDL (‘’good’’ cholesterol), as well as an increase in ‘’happiness’’ hormones (serotonin and dopamine) was observed. Moreover, it is important to note that the liver enzymes ALT, ALP, and GGT did not significantly increase in consumer groups, although alcohol concentration was relatively high in all the wines.

Our data support the claim that the protective effect of moderate wine consumption can be attributed to its bioactive compound content; moreover, this relatively short-term study suggests that moderate wine consumption of 200 mL per day can be considered as a component of a balanced diet and healthy lifestyle regarding the investigated parameters.

## Figures and Tables

**Table 3:** Concentrations of HPLC individual phenols (mg/L) of young and aged Malvazija istarska and Pošip wines.

Phenols	Malvazija Istarska		Pošip	
	Young	Aged	Young	Aged
**Phenolic acids, flavonols, and flavanonols**				
Gallic acid	28.53 ± 0.01 ^b^	4.64 ± 0.02 ^c^	3.08 ± 0.01 ^d^	30.93 ± 0.03 ^a^
Protocatechuic acid	0.64 ± 0.02 ^c^	4.56 ± 0.05 ^a^	1.02 ± 0.17 ^b^	1.03 ± 0.04 ^b^
*p*-Hydroxybenzoic acid	0.67 ± 0.01 ^b^	1.48 ± 0.05 ^a^	0.29 ± 0.01 ^c^	0.23 ± 0.01 ^d^
Syringic acid	0.43 ± 0.00 ^a^	0.16 ± 0.00 ^c^	0.14 ± 0.01 ^d^	0.28 ± 0.00 ^b^
*cis*-Caftaric acid	0.71 ± 0.00 ^a^	0.35 ± 0.00 ^c^	0.50 ± 0.00 ^b^	0.32 ± 0.00 ^d^
*trans*-Caftaric acid	7.17 ± 0.00 ^d^	45.92 ± 0.12 ^b^	21.07 ± 0.01 ^c^	46.70 ± 0.04 ^a^
Caffeic acid	11.71 ± 0.00 ^a^	8.13 ± 0.04 ^c^	1.22 ± 0.01 ^d^	10.61 ± 0.01 ^b^
*p*-Coumaric acid	1.51 ± 0.00 ^d^	2.79 ± 0.00 ^b^	1.79 ± 0.00 ^c^	3.91 ± 0.02 ^a^
Ferulic acid	0.95 ± 0.00 ^a^	n.d.	0.54 ± 0.00 ^c^	0.79 ± 0.01 ^b^
Taxifolin	0.38 ± 0.00 ^d^	0.50 ± 0.00 ^c^	0.90 ± 0.01 ^a^	0.70 ± 0.01 ^b^
Quercetin	0.17 ± 0.00 ^d^	0.78 ± 0.04 ^c^	2.37 ± 0.01 ^a^	1.21 ± 0.00 ^b^
Total phenolic acids, flavonols, and flavanonols	52.88 ± 0.03 ^c^	69.33 ± 0.17 ^b^	32.92 ± 0.17 ^d^	96.71 ± 0.11 ^a^
**Flavan-3-ols**				
Procyanidin B1	0.56 ± 0.07 ^c^	0.98 ± 0.09 ^b^	1.89 ± 0.19 ^a^	0.94 ± 0.27 ^b^
Procyanidin B3	3.60 ± 0.01 ^b^	1.14 ± 0.07 ^d^	4.68 ± 0.03 ^a^	1.58 ± 0.07 ^c^
(+)-Catechin	2.96 ± 0.32 ^d^	5.06 ± 0.10 ^c^	8.08 ± 0.30 ^a^	6.16 ± 0.79 ^b^
Procyanidin B2	0.38 ± 0.05 ^c^	0.53 ± 0.06 ^b^	0.96 ± 0.08 ^a^	0.50 ± 0.08 ^bc^
(−)-Epicatechin	3.40 ± 0.26 ^a^	0.99 ± 0.01 ^c^	3.61 ± 0.09 ^a^	2.64 ± 0.21 ^b^
Procyanidin C1	0.36 ± 0.01 ^b^	0.35 ± 0.01 ^b^	0.29 ± 0.01 ^c^	0.42 ± 0.01 ^a^
Total flavan-3-ols	11.26 ± 0.66 ^b^	9.05 ± 0.32 ^c^	19.52 ± 0.69 ^a^	12.24 ± 1.38 ^b^
**Stilbenes**				
*trans*-Piceid	n.d.	n.d.	0.35 ± 0.08 ^b^	0.85 ± 0.08 ^a^
Piceatannol	0.09 ± 0.01 ^ab^	0.08 ± 0.00 ^b^	0.10 ± 0.02 ^a^	0.06 ± 0.00 ^c^
Resveratrol	0.12 ± 0.00 ^b^	0.12 ± 0.00 ^b^	0.12 ± 0.01 ^b^	0.17 ± 0.00 ^a^
*cis*-Piceid	0.18 ± 0.00 ^c^	0.30 ± 0.02 ^b^	0.39 ± 0.03 ^a^	0.40 ± 0.01 ^a^
Total stilbenes	0.39 ± 0.01 ^c^	0.51 ± 0.02 ^c^	0.96 ± 0.14 ^b^	1.48 ± 0.08 ^a^
Total HPLC phenols	64.54 ± 0.63 ^c^	78.89 ± 0.22 ^b^	53.4 ± 0.75 ^d^	110.42 ± 1.44 ^a^

n.d.—not detected; young white wines produced without maceration and aged white wines produced with 7 days maceration followed by maturation in oak barrels. Each value represents the mean ± standard deviation; different lowercase superscript letters represent statistically significant differences between wine samples at *p* < 0.05 obtained by one-way ANOVA and least significant difference (LSD) test.

**Table 4:** Concentrations of HPLC individual phenols (mg/L) of young and aged Teran and Plavac mali wines.

Phenols	Teran		Plavac Mali	
	Young	Aged	Young	Aged
**Phenolic acids, flavonols, and flavanonols**				
Gallic acid	42.69 ± 0.05 ^d^	157.92 ± 0.31 ^a^	52.58 ± 0.07 ^c^	94.44 ± 0.11 ^b^
Protocatechuic acid	5.16 ± 0.09 ^c^	7.37 ± 0.07 ^b^	5.27 ± 0.18 ^c^	7.75 ± 0.29 ^a^
*p*-Hydroxybenzoic acid	1.26 ± 0.02 ^c^	1.68 ± 0.05 ^b^	1.28 ± 0.01 ^c^	2.05 ± 0.04 ^a^
Syringic acid	10.83 ± 0.02 ^a^	9.16 ± 0.01 ^b^	6.00 ± 0.05 ^d^	7.43 ± 0.05 ^c^
*cis*-Caftaric acid	0.46 ± 0.00 ^a^	0.29 ± 0.01 ^c^	0.41 ± 0.00 ^b^	n.d.
*trans*-Caftaric acid	55.95 ± 0.04 ^b^	61.79 ± 0.10 ^a^	8.13 ± 0.01 ^d^	26.18 ± 0.03 ^c^
Caffeic acid	3.29 ± 0.00 ^d^	4.24 ± 0.06 ^c^	8.80 ± 0.00 ^a^	6.72 ± 0.01 ^b^
*p*-Coumaric acid	3.33 ± 0.06 ^b^	2.04 ± 0.03 ^d^	2.81 ± 0.02 ^c^	5.17 ± 0.04 ^a^
Ferulic acid	0.39 ± 0.00 ^c^	0.37 ± 0.01 ^c^	1.01 ± 0.05 ^a^	0.54 ± 0.03 ^b^
Taxifolin	0.56 ± 0.01 ^c^	0.38 ± 0.01 ^d^	1.74 ± 0.01 ^b^	3.15 ± 0.02 ^a^
Quercetin 3-glucoside + Quercetin 3-glucuronide	n.d.	0.39 ± 0.01 ^b^	7.02 ± 0.24 ^a^	0.52 ± 0.01 ^b^
Myricetin	4.35 ± 0.09 ^b^	2.67 ± 0.03 ^d^	2.86 ± 0.00 ^c^	5.36 ± 0.00 ^a^
Quercetin	19.74 ± 0.04 ^c^	35.21 ± 0.45 ^a^	20.81 ± 0.17 ^b^	19.11 ± 0.13 ^d^
Total phenolic acids, flavonols, and flavanonols	148.00 ± 0.29 ^c^	283.53 ± 0.75 ^a^	118.72 ± 0.37 ^d^	178.41 ± 0.26 ^b^
**Flavan-3-ols**				
Procyanidin B1	10.68 ± 0.01 ^d^	40.49 ± 0.35 ^a^	29.41 ± 0.01 ^b^	27.70 ± 0.06 ^c^
Procyanidin B3	4.53 ± 0.01 ^c^	14.51 ± 0.23 ^a^	7.99 ± 0.01 ^b^	7.77 ± 0.08 ^b^
(+)-Catechin	23.52 ± 0.01 ^d^	44.30 ± 0.32 ^a^	26.80 ± 0.05 ^c^	32.18 ± 0.12 ^b^
Procyanidin B2	11.91 ± 0.01 ^d^	34.32 ± 0.32 ^a^	18.03 ± 0.11 ^b^	17.39 ± 0.05 ^c^
(−)-Epicatechin	18.40 ± 0.02 ^b^	27.7 ± 0.29 ^a^	13.83 ± 0.05 ^d^	14.69 ± 0.04 ^c^
Procyanidin C1	1.37 ± 0.03 ^d^	7.44 ± 0.14 ^a^	4.60 ± 0.02 ^b^	3.03 ± 0.04 ^c^
Total flavan-3-ols	70.41 ± 0.01 ^d^	168.75 ± 1.38 ^a^	100.66 ± 0.17 ^c^	102.76 ± 1.22 ^b^
**Stilbenes**				
*trans*-Piceid	4.42 ± 0.02 ^d^	8.98 ± 0.02 ^b^	5.68 ± 0.03 ^c^	9.05 ± 0.03 ^a^
Piceatannol	0.17 ± 0.00 ^b^	0.14 ± 0.01 ^c^	0.06 ± 0.00 ^d^	0.19 ± 0.00 ^a^
Resveratrol	0.72 ± 0.00 ^b^	0.45 ± 0.01 ^c^	0.69 ± 0.02 ^b^	0.82 ± 0.00 ^a^
*cis*-Piceid	2.97 ± 0.01 ^d^	5.58 ± 0.04 ^a^	4.23 ± 0.01 ^b^	3.46 ± 0.04 ^c^
Total stilbenes	8.28 ± 0.04 ^d^	15.15 ± 0.06 ^a^	10.66 ± 0.06 ^c^	13.52 ± 0.07 ^b^
**Anthocyanins**				
Delphinidin 3-glucoside	0.52 ± 0.01 ^d^	1.15 ± 0.02 ^b^	1.55 ± 0.02 ^a^	1.03 ± 0.00 ^c^
Cyanidin 3-glucoside	0.08 ± 0.00 ^d^	0.21 ± 0.01 ^a^	0.17 ± 0.00 ^b^	0.11 ± 0.00 ^c^
Petunidin 3-glucoside	0.54 ± 0.01 ^d^	0.93 ± 0.02 ^b^	1.63 ± 0.02 ^a^	0.79 ± 0.00 ^c^
Pelargonidin 3-glucoside	0.15 ± 0.01 ^a^	0.09 ± 0.00 ^b^	0.16 ± 0.00 ^a^	0.07 ± 0.00 ^c^
Peonidin 3-glucoside	0.50 ± 0.01 ^d^	0.84 ± 0.02 ^b^	1.70 ± 0.01 ^a^	0.58 ± 0.00 ^c^
Malvidin 3-glucoside	9.09 ± 0.14 ^c^	6.54 ± 0.15 ^d^	26.30 ± 0.14 ^a^	10.26 ± 0.04 ^b^
Peonidin 3-acetylglucoside	0.17 ± 0.01 ^c^	0.37 ± 0.03 ^a^	0.36 ± 0.01 ^a^	0.25 ± 0.00 ^b^
Malvidin 3-acetylglucoside	1.26 ± 0.02 ^c^	1.10 ± 0.02 ^d^	1.75 ± 0.01 ^a^	1.30 ± 0.01 ^b^
Peonidin 3-cumarylglucoside	0.07 ± 0.01 ^c^	0.11 ± 0.01 ^b^	0.20 ± 0.01 ^a^	0.11 ± 0.00 ^b^
Malvidin 3-cumarylglucoside	0.93 ± 0.04 ^c^	0.82 ± 0.03 ^d^	1.82 ± 0.05 ^a^	1.65 ± 0.01 ^b^
Total anthocyanins	13.31 ± 0.24 ^c^	12.15 ± 0.30 ^d^	35.64 ± 0.25 ^a^	16.15 ± 0.05 ^b^
Total HPLC phenols	240.00 ± 0.42 ^d^	479.58 ± 0.67 ^a^	265.67 ± 0.53 ^c^	310.84 ± 0.39 ^b^

n.d.—not detected; young red wines produced with standard 7–8 days maceration and aged red wines produced with prolonged maceration (15–30 days) and maturation in oak barrels. Each value represents the mean ± standard deviation; different lowercase superscript letters represent statistically significant differences between wine samples at *p* < 0.05 obtained by one-way ANOVA and least significant difference (LSD) test.

**Table 5:** Concentration (mg/L) of macro- and microelements of young and aged Malvazija istarska and Pošip wines.

Macro- and Microelements (mg/L)	Malvazija Istarska		Pošip	
	Young	Aged	Young	Aged
K	888.0 ± 1.73 ^b^	778.37 ± 1.66 ^d^	894.1 ± 2.70 ^a^	785.87 ± 1.86 ^c^
Ca	71.28 ± 0.84 ^b^	62.76 ± 0.58 ^d^	82.42 ± 0.43 ^a^	70.11 ± 0.1 ^c^
Mg	82.4 ± 0.20 ^d^	93.6 ± 0.20 ^c^	95.27 ± 0.35 ^b^	98.47 ± 0.25 ^a^
Na	39.68 ± 0.05 ^a^	37.56 ± 0.05 ^c^	38.32 ± 0.08 ^b^	36.62 ± 0.51 ^d^
Total macroelements (mg/L)	1081.36 ± 2.14 ^b^	972.29 ± 2.37 ^d^	1110.1 ± 2.85 ^a^	991.07 ± 1.22 ^c^
Al	1.27 ± 0.03 ^a^	1.04 ± 0.01 ^c^	1.30 ± 0.02 ^a^	1.11 ± 0.02 ^b^
Cu	0.040 ± 0.001 ^b^	0.035 ± 0.001 ^c^	0.042 ± 0.002 ^a^	0.040 ± 0.001 ^b^
Fe	1.88 ± 0.02 ^a^	1.54 ± 0.01 ^c^	1.81 ± 0.01 ^b^	1.27 ± 0.02 ^d^
Mn	0.737 ± 0.002 ^b^	0.631 ± 0.001 ^c^	0.797 ± 0.001 ^a^	0.614 ± 0.001 ^d^
Total microelements (mg/L)	3.93 ± 0.01 ^a^	3.25 ± 0.02 ^b^	3.95 ± 0.03 ^a^	3.04 ± 0.03 ^c^

Each value represents the mean ± standard deviation; young white wines produced without maceration and aged white wines produced with 7 days maceration followed by maturation in oak barrels. Different lowercase superscript letters represent statistically significant differences between wine samples at *p* < 0.05 obtained by one-way ANOVA and least significant difference (LSD) test.

**Table 6:** Concentration (mg/L) of macro- and microelements in young and aged Teran and Plavac mali wines.

Macro- and Microelements (mg/L)	Teran		Plavac Mali	
	Young	Aged	Young	Aged
K	864.83 ± 0.8 ^b^	867.24 ± 1.61 ^ab^	869.73 ± 2.53 ^a^	869.03 ± 1.81 ^a^
Ca	165.23 ± 1.08 ^a^	160.4 ± 1.08 ^b^	142.3 ± 1.15 ^c^	142.4 ± 0.61 ^c^
Mg	119.07 ± 1.01 ^b^	121.33 ± 0.70 ^a^	114.67 ± 0.60 ^c^	115.07 ± 1.06 ^c^
Na	7.56 ± 0.01 ^b^	7.38 ± 0.01 ^a^	7.12 ± 0.01 ^c^	7.1 ± 0.01 ^d^
Total macroelements (mg/L)	1156.69 ± 2.87 ^a^	1156.35 ± 3.38 ^a^	1133.82 ± 4.25 ^b^	1133.61 ± 2.94 ^b^
Al	2.28 ± 0.02 ^a^	2.07 ± 0.01 ^b^	1.96 ± 0.01 ^c^	1.78 ± 0.01 ^d^
Cu	0.058 ± 0.002 ^d^	0.062 ± 0.001 ^c^	0.074 ± 0.002 ^a^	0.067 ± 0.001 ^b^
Fe	9.99 ± 0.01 ^a^	9.55 ± 0.01 ^b^	8.45 ± 0.01 ^c^	8.28 ± 0.01 ^d^
Mn	1.50 ± 0.01 ^a^	1.48 ± 0.01 ^b^	1.34 ± 0.00 ^c^	1.32 ± 0.02 ^c^
Total microelements (mg/L)	13.83 ± 0.03 ^a^	13.16 ± 0.02 ^b^	11.82 ± 0.02 ^c^	11.45 ± 0.02 ^d^

Each value represents the mean ± standard deviation; young red wines produced with standard 7–8 days maceration and aged red wines produced with prolonged maceration (15–30 days) and maturation in oak barrels. Different lowercase superscript letters represent statistically significant differences between wine samples at *p* < 0.05 obtained by one-way ANOVA and least significant difference (LSD) test.

**Table 7:** Concentration (μg/L) of vitamins in young and aged Malvazija istarska and Pošip wine.

Vitamins (μg/L)	Malvazija Istarska		Pošip	
	Young	Aged	Young	Aged
Vitamin C	n.d.	n.d.	n.d.	n.d.
Vitamin B1	4.23 ± 0.21 ^a^	2.10 ± 0.00 ^c^	3.80 ± 0.00 ^b^	1.90 ± 0.00 ^d^
Vitamin B2	62.93 ± 0.25 ^a^	31.43 ± 0.49 ^c^	62.10 ± 0.10 ^b^	29.53 ± 0.45 ^d^
Vitamin B3	501.43 ± 5.26 ^a^	383.57 ± 4.00 ^c^	485.23 ± 2.95 ^b^	277.73 ± 2.70 ^d^
Vitamin B6	165.73 ± 2.55 ^a^	104.3 ± 2.23 ^c^	157.6 ± 2.51 ^b^	96.47 ± 1.50 ^d^
Total vitamins (μg/L)	734.33 ± 2.77 ^a^	521.4 ± 6.64 ^c^	708.74 ± 5.55 ^b^	405.63 ± 1.66 ^d^

n.d.—not detected. Each value represents the mean ± standard deviation; young white wines produced without maceration and aged white wines produced with 7 days maceration followed by maturation in oak barrels. Different lowercase superscript letters represent statistically significant differences between wine samples at *p* < 0.05 obtained by one-way ANOVA and least significant difference (LSD) test.

**Table 8:** Concentration (μg/L) of vitamins in young and aged Teran and Plavac mali wines.

Vitamins (μg/L)	Teran		Plavac Mali	
	Young	Aged	Young	Aged
Vitamin C	n.d.	n.d.	n.d.	n.d.
Vitamin B1	25.47 ± 0.70 ^a^	16.00 ± 0.20 ^b^	13.98 ± 0.25 ^c^	10.15 ± 0.90 ^d^
Vitamin B2	329.13 ± 1.95 ^a^	217.27 ± 4.25 ^b^	221.5 ± 1.67 ^b^	127.83 ± 2.52 ^c^
Vitamin B3	807.73 ± 4.08 ^a^	643.37 ± 3.85 ^b^	597.2 ± 2.67 ^c^	306.2 ± 5.71 ^d^
Vitamin B6	349.5 ± 2.05 ^a^	227.57 ± 2.73 ^c^	239.3 ± 0.95 ^b^	147.1 ± 3.68 ^d^
Total vitamins (μg/L)	1511.83 ± 8.76 ^a^	1071.98 ± 0.2 ^c^	1104.2 ± 10.9 ^b^	591.28 ± 7.77 ^d^

n.d.—not detected. Each value represents the mean ± standard deviation; young red wines produced with standard 7–8 days maceration and aged red wines produced with prolonged maceration (15–30 days) and maturation in oak barrels. Different lowercase superscript letters represent statistically significant differences between wine samples at *p* < 0.05 obtained by one-way ANOVA and least significant difference (LSD) test.

**Table 9:** Changes in different medical examination parameters after the consumption period in eight consumer groups and one non-consumer group of study participants.

Medical Examination Parameters	Non-Consumer Control Group	Consumer Groups								
		Malvazija Istarska		Pošip		Teran		Plavac Mali		
		Young	Aged	Young	Aged	Young	Aged	Young	Aged	
Weight (kg)	−0.58 ± 1.67 ^bc^	0.66 ± 0.91 ^ab^	−0.06 ± 0.76 ^abc^	−1.03 ± 1.81 ^c^	1.0 ± 1.3 ^a^	0.01 ± 1.28 ^abc^	0.27 ± 1.92 ^abc^	0.01 ± 1.43 ^abc^	−0.4 ± 0.64 ^abc^	
Body mass index BMI (kg/m^2^)	−0.2 ± 0.6 ^ab^	0.31 ± 0.37 ^a^	0.15 ± 0.45 ^ab^	−0.36 ± 0.72 ^ab^	0.32 ± 0.38 ^a^	0.07 ± 0.6 ^ab^	0.14 ± 0.62 ^ab^	−0.06 ± 0.53 ^ab^	−0.17 ± 0.37 ^ab^	
Waistline (cm)	−2.6 ± 6.47 ^bd^	5.75 ± 5.6 ^ab^	4.0 ± 5.1 ^abc^	7.86 ± 4.14 ^a^	3.29 ± 3.64 ^abc^	−0.57 ± 5.74 ^bc^	2.33 ± 6.44 ^abc^	0.71 ± 6.29 ^b^	1.29 ± 6.7 ^b^	
Hip width (cm)	1.3 ± 3.65	1.25 ± 2.76	0.71 ± 3.45	3.71 ± 5.38	1.14 ± 3.24	0.86 ± 2.19	2.0 ± 3.41	1.71 ± 1.8	0.57 ± 2.57	n.s.
Heart Rate/min	−3.1 ± 9.17	−1.5 ± 8.26	−1.43 ± 9.29	4.57 ± 12.26	−1.43 ± 9.14	−1.43 ± 10.05	−2.0 ± 4.2	1.43 ± 4.58	0.29 ± 10.16	n.s.
RR Systolic mmHg	−1.5 ± 14.35 ^ab^	6.25 ± 11.88 ^a^	2.14 ± 5.67 ^ab^	1.43 ± 9.88 ^ab^	−2.86 ± 6.99 ^ab^	−0.71 ± 10.58 ^ab^	8.33 ± 7.53 ^a^	2.14 ± 7.56 ^ab^	−6.43 ± 14.06 ^b^	
RR Diastolic mmHg	−2 ± 11.1	0.63 ± 7.76	0.71 ± 5.35	−2.14 ± 4.88	−1.43 ± 8.02	−0.71 ± 4.5	−0.83 ± 8.01	1.423 ± 6.27	−3.57 ± 6.9	n.s.

n.s.—difference between values within each row is not statistically significant. Each value is expressed as mean ± standard deviation; different lowercase superscript letters represent statistically significant differences between groups at *p* < 0.05 obtained by one-way ANOVA and least significant difference (LSD) test.

**Table 10:** Changes in different blood parameters between the first and the second measurement in eight consumer groups and one non-consumer group of study participants.

Blood Parameters	Non-Consumer Control Group	Consumer Groups							
		Malvazija Istarska		Pošip		Teran		Plavac Mali	
		Young	Aged	Young	Aged	Young	Aged	Young	Aged
Erythrocytes [1 × 10^12^]/L	−0.03 ± 0.22	−0.13 ± 0.13	0.02 ± 0.23	−0.01 ± 0.12	0.07 ± 0.13	−0.08 ± 0.25	0.08 ± 0.17	0.02 ± 0.15	−0.08 ± 0.25
Hemoglobin (g/L)	−1.2 ± 5.22 ^ab^	−3.13 ± 3.23 ^ab^	0.71 ± 7.61 ^ab^	−1.57 ± 4.76 ^ab^	2.0 ± 4.16 ^a^	−4.14 ± 7.29 ^b^	1.83 ± 4.26 ^ab^	0.14 ± 4.53 ^ab^	−1.86 ± 7.1 ^ab^
RDW (%)	0.03 ± 0.3 ^b^	0.06 ± 0.23 ^b^	0.04 ± 0.37 ^b^	−0.09 ± 0.28 ^b^	−0.03 ± 0.26 ^b^	0.07 ± 0.53 ^b^	0.62 ± 1.16 ^a^	0.1 ± 0.31 ^ab^	0.09 ± 0.43 ^b^
Thrombocytes ([1 × 10^9^]/L)	−10.9 ± 13.2 ^bc^	−4.6 ± 15.57 ^abc^	7.14 ± 18.76 ^ab^	5.86 ± 18.74 ^ab^	−2.86 ± 22.56 ^abc^	12.0 ± 26.7 ^a^	−17.83 ± 27.37 ^c^	−3.0 ± 14.8 ^abc^	1.29 ± 16.45 ^abc^
MPV (fL)	0.08 ± 0.24 ^a^	0 ± 0.33 ^a^	0.07 ± 0.29 ^a^	−0.36 ± 0.37 ^b^	−0.04 ± 0.4 ^ab^	−0.03 ± 0.33 ^ab^	0.22 ± 0.27 ^a^	0.19 ± 0.16 ^a^	0.2 ± 0.34 ^a^
Leukocytes ([1 × 10^9^]/L)	−0.22 ± 0.72 ^b^	0.48 ± 0.74 ^ab^	0.39 ± 1.32 ^ab^	0.63 ± 0.77 ^a^	−0.07 ± 0.78 ^ab^	−0.13 ± 0.75 ^ab^	−0.13 ± 0.41 ^ab^	0.21 ± 0.4 ^ab^	0.06 ± 0.75 ^ab^
Glucose (mmol/L)	−0.16 ± 0.33	−0.29 ± 0.36	−0.26 ± 0.61	0.1 ± 0.44	0.06 ± 0.4	−0.3 ± 0.61	−0.32 ± 0.38	0.13 ± 0.36	−0.3 ± 0.8
Urea (mmol/L)	−0.35 ± 1.05 ^b^	0.88 ± 1.06 ^a^	0.49 ± 0.53 ^ab^	−0.19 ± 1.02 ^b^	0.56 ± 1.42 ^ab^	−0.18 ± 0.71 ^b^	−0.02 ± 1.07 ^ab^	1.02 ± 1.01 ^a^	0.35 ± 0.81 ^ab^
Creatinine (µmol/L)	3.3 ± 5.6 ^ab^	3.25 ± 4.65 ^ab^	8.14 ± 4.14 ^a^	4.29 ± 6.87 ^ab^	3.43 ± 5.32 ^ab^	3.23 ± 7.43 ^ab^	1.0 ± 8.3 ^b^	5.29 ± 3.40 ^ab^	2.57 ± 6.02 ^ab^
eGFR CKD-EPI	−4.3 ± 8.06	−4.25 ± 4.65	−8.29 ± 5.38	−5.43 ± 8.42	−3.57 ± 6.24	−4.43 ± 9.38	−1.17 ± 11.48	−5.0 ± 3.83	−2.87 ± 6.47
Na (mmol/L)	0.0 ± 1.94 ^b^	0.75 ± 1.75 ^ab^	0.29 ± 1.98 ^ab^	1.0 ± 2.65 ^ab^	1.29 ± 2.29 ^ab^	1.71 ± 3.3 ^ab^	−0.17 ± 1.94 ^b^	2.5 ± 1.81 ^a^	1.43 ± 1.62 ^ab^
K (mmol/L)	0.02 ± 0.31	−0.05 ± 0.27	0.14 ± 0.53	−0.03 ± 0.4	0.16 ± 0.17	0.19 ± 0.33	0.02 ± 0.19	0.2 ± 0.23	−0.04 ± 0.29
Bilirubin (µmol/L)	−0.5 ± 4.72	0.25 ± 5.68	0.86 ± 4.38	0.57 ± 2.37	−1.86 ± 3.24	−1.29 ± 5.41	0.33 ± 2.94	0.0 ± 2.3	−3.29 ± 6.99
Uric acid (µmol/L)	8.9 ± 39.04 ^a^	−25 ± 23.11 ^b^	0.43 ± 18.08 ^ab^	−0.29 ± 39.85 ^ab^	10.57 ± 33.29 ^a^	−7.43 ± 43.06 ^ab^	−4.12 ± 43.28 ^ab^	−0.86 ± 30.09 ^ab^	0.86 ± 27.53 ^ab^
AST (U/L)	0.8 ± 3.36	0.75 ± 2.82	−0.14 ± 4.1	0.14 ± 4.14	0.14 ± 1.95	0.29 ± 3.99	1.16 ± 1.17	2.0 ± 2.45	−0.43 ± 2.3
ALT (U/L)	1.3 ± 11.94 ^b^	3.75 ± 2.76 ^b^	1.29 ± 7.25 ^b^	1.14 ± 5.46 ^b^	5.57 ± 3.41 ^ab^	0.57 ± 8.66 ^b^	4.0 ± 1.79 ^ab^	16.0 ± 29.82 ^a^	0.57 ± 3.82 ^b^
ALP (U/L)	0.3 ± 5.44 ^ab^	0.87 ± 3.94 ^ab^	−3.0 ± 5.92 ^ab^	1.29 ± 4.11 ^ab^	−0.57 ± 3.36 ^ab^	−4.29 ± 9.41 ^b^	0.16 ± 5.95 ^ab^	0.43 ± 8.1 ^ab^	3.0 ± 7.16 ^a^
GGT (U/L)	−0.9 ± 9.76 ^b^	−0.5 ± 4.14 ^b^	2.0 ± 2.94 ^b^	0.15 ± 6.91 ^b^	2.43 ± 1.72 ^b^	−0.29 ± 3.9 ^b^	2.33 ± 2.8 ^ab^	18.0 ± 40.89 ^a^	0.86 ± 1.77 ^b^
Iron (µmol/L)	−1.8 ± 7.74	−0.63 ± 3.85	−0.86 ± 8.17	−0.29 ± 2.69	0.71 ± 7.16	0.29 ± 4.79	1.83 ± 8.04	0.14 ± 4.45	−4.57 ± 10.26
UIBC (µmol/L)	1.2 ± 7.48	1.63 ± 6.39	2.93 ± 7.32	0.57 ± 3.1	0.861 ± 8.51	0.57 ± 5.62	−1.5 ± 11.45	1.43 ± 4.86	4.29 ± 8.48
Feritin (µg/L)	−0.9 ± 11.43	−0.88 ± 28.26	−9.86 ± 26.31	−6.14 ± 18.95	−9.57 ± 14.18	−19.71 ± 24.07	−3.83 ± 16.67	−14.0 ± 19.02	−14.43 ± 20.98
Cholesterol (mmol/L)	−0.09 ± 0.59 ^ab^	−0.04 ± 0.7 ^ab^	−0.19 ± 0.51 ^ab^	−0.04 ± 0.43 ^ab^	0.37 ± 0.61 ^a^	0.23 ± 0.5 ^a^	0.23 ± 0.4 ^a^	−0.06 ± 0.6 ^ab^	−0.56 ± 0.65 ^b^
HDL (mmol/L)	0.04 ± 0.15	0.15 ± 0.19	0.11 ± 0.22	0.07 ± 0.11	0.14 ± 0.1	0.16 ± 0.17	0.2 ± 0.14	0.07 ± 0.11	0.06 ± 0.17
LDL (mmol/L)	−0.15 ± 0.61 ^abc^	−0.23 ± 0.5 ^abc^	−0.34 ± 0.51 ^bc^	−0.90 ± 0.51 ^abc^	0.24 ± 0.52 a	0.06 ± 0.54 ^ab^	−0.08 ± 0.33 ^abc^	−0.2 ± 0.4 ^abc^	−0.6 ± 0.5 ^c^
Triglycerides (mmol/L)	0.16 ± 0.94	0.125 ± 0.25	0.1143 ± 0.47	−0.03 ± 0.66	−0.01 ± 0.26	0.01 ± 0.45	0.25 ± 0.38	0.16 ± 0.79	−0.06 ± 0.19
hs-CRP (mg/L)	−0.62 ± 1.57 ^b^	0 ± 0.71 ab	−0.07 ± 0.24 ^ab^	0.36 ± 1.03 ^ab^	−0.16 ± 0.42 ^b^	−0.4 ± 0.68 ^b^	0.23 ± 0.7 ^ab^	1.63 ± 4.45 ^a^	0.04 ± 0.26 ^ab^
SEROTONIN (ng/mL)	2.2 ± 44.16 ^ab^	21.36 ± 115.12 ^ab^	35.86 ± 36.34 ^a^	12.14 ± 64.67 ^ab^	−11.85 ± 23.6 ^ab^	−8.29 ± 50.95 ^ab^	−24.7 ± 29.6 ^ab^	16.57 ± 36.13 ^ab^	−33.14 ± 59.34 ^b^
DOPAMINE (ng/mL)	0.51 ± 2.01 ^ab^	6.05 ± 9.17 ^a^	−0.95 ± 3.17 ^b^	0.17 ± 0.47 ^ab^	−0.69 ± 2.59 ^b^	0.22 ± 5.87 ^ab^	1.79 ± 11.05 ^ab^	−2.81 ± 9.04 ^b^	−0.84 ± 5.35 ^b^

Each value represents the mean ± standard deviation; different lowercase superscript letters represent statistically significant differences between groups at *p* < 0.05 obtained by one-way ANOVA and least significant difference (LSD) test.

## Data Availability

Data is contained within the article.
